# Systematic characterization of cross-source miRNA biomarkers in prostate cancer with computational-experimental integrated analysis

**DOI:** 10.3389/fcell.2025.1605297

**Published:** 2025-09-25

**Authors:** Huimin Lu, Wenjin Li, Zhongxin Huang, Libo Chen, Mingyong Li, Weiming Deng

**Affiliations:** ^1^ Department of Urology and Andrology, Sir Run Run Shaw Hospital, Zhejiang University School of Medicine, Hangzhou, China; ^2^ Department of Nutrition, The Second Affiliated Hospital, Hengyang Medical School, University of South China, Hengyang, China; ^3^ Department of Urology, The First Affiliated Hospital, Hengyang Medical School, University of South China, Hengyang, China

**Keywords:** microRNA, prostate cancer, biomarkers, bioinformatics, regulatory network

## Abstract

**Purpose:**

Prostate cancer (PCa) is occult and remains largely incurable once it metastasizes. Our research aims to identify the key miRNAs and construct miRNA–mRNA networks for PCa.

**Methods:**

The microarray dataset GSE112264, consisting of 1,591 male serum samples, and tissue miRNA data from TCGA, including 497 prostate cancer and 52 normal samples, were included in the analysis. Differentially expressed miRNAs (DE-miRNAs) were detected, and miRTarBase was used to predict the common target genes. Then, Gene Ontology (GO) and Kyoto Encyclopedia of Genes and Genomes (KEGG) pathway enrichment analyses were performed for the target genes. The protein–protein interaction (PPI) network, which revealed the top 10 hub genes, was constructed using the Search Tool for the Retrieval of Interacting Genes/Proteins (STRING) and Cytoscape. The expression of the potential hub genes was examined using the UALCAN database. Finally, GSE112264, TCGA datasets, and clinical samples were used to verify the consistency of miRNA expressions in serum and tissue.

**Results:**

A total of 948 target genes of the two overlapped downregulated miRNAs (miR-146a-3p and miR-136-3p) were predicted. Functional enrichment analysis indicated that significant DE-miRNAs were related to PCa-related pathways, such as protein binding, the mammalian target of rapamycin (mTOR) signaling pathway, and porphyrin and chlorophyll metabolisms. Four hub genes were identified from the PPI network, namely, NSF, HIST2H2BE, IGF2R, and CADM1, and verified to be aberrantly expressed in the UALCAN database. Experiment results indicated that only miR-136-3p was markedly reduced in both serum and tissue.

**Conclusion:**

In this study, we established the miRNA–mRNA network, offering potential PCa targets.

## Introduction

Prostate cancer (PCa) is the second most common cancer among men worldwide. Localized PCa is usually treated with surgery and radiation therapy, which are ineffective at the distant metastasis stage (Sartor and de Bono, 2018). PCa is characterized by distant metastasis, which most commonly occurs in the bones, liver, lungs, and brain (Kfoury et al., 2021). Metastatic and advanced PCa induces drug resistance to current therapies, which contributes to the poor prognosis. Nearly 80% of patients treated with androgen deprivation therapy finally become unresponsive, resulting in a median survival of only 14 months (Shafi et al., 2013). Therefore, the identification of pathophysiological mechanism and diagnostic biomarkers needs further investigation.

Recently, developments in microRNAs (miRNAs), which are endogenous single-stranded noncoding RNAs regulating gene expression post-transcriptionally, provided new insights into the pathogenesis of cancer (Lu and Rothenberg, 2018; Kanavarioti et al., 2024; Wu et al., 2024; Hor et al., 2023). Different types of tumors can be regulated by miRNAs, which function as either tumor suppressors or oncogenes, such as miR-21, which has both oncogenic and onco-suppressor functions (Hashemi et al., 2023). MiRNAs involved in PCa tumorigenesis are usually found to be deregulated, influencing many processes at the molecular and cellular levels (Padmyastuti et al., 2023; Slabáková et al., 2021; Wang et al., 2024; Lu et al., 2023; Armstrong et al., 2024). For example, miR-24, functioning as a cancer suppressor, is frequently downregulated in PCa cells (Cheng et al., 2021). Another oncogene, miR-888, promotes PCa growth by suppressing retinoblastoma-like protein 1, which can directly bind to the transcriptional factor E2F and regulate cell cycle progression from the G1 to S phase (Hasegawa et al., 2018). Furthermore, a study reports an exovesicle-derived miR-20a-5p, which can regulate PCa cell proliferation and inflammation through the RORA gene (Sánchez et al., 2024). The potential utility of miRNA as biomarkers has been widely reported in the past decade (Fabris et al., 2016). Despite that, there are very few studies analyzing the miRNA–mRNA regulatory network in PCa. Research on the role of miRNA in PCa is crucial for early diagnosis and effective treatment.

In this research, we screened out differentially expressed miRNAs (DE-miRNAs) in serum and tissue samples of PCa using bioinformatics methods (Barrett et al., 2013; Blum et al., 2018). As predicted by miRTarBase, miR-146a-3p and miR-136-3p are two of the most downregulated miRNAs. Gene Ontology (GO) and Kyoto Encyclopedia of Genes and Genomes (KEGG) pathway enrichment analyses were used for detecting potential biological functions of the 948 target genes through Database for Annotation, Visualization, and Integrated Discovery (DAVID). We also developed the protein–protein interaction (PPI) network using Cytoscape to reveal regulatory mechanisms of miRNA–mRNA networks. The expression of the top 10 target genes was further validated using the UALCAN database. We further validated our findings using the UALCAN database by obtaining serum samples from PCa and benign prostatic hypertrophy patients and measuring expression levels of miR-146a-3p and miR-136-3p in these samples. Only miR-136-3p maintained consistency in the serum and tissue. In this study, we aim to identify PCa-associated miRNAs through various bioinformatic analyses and validate the consistency of miR-136-3p expression between serum and tissue samples. For this reason, our findings may provide a simpler diagnosis of PCa using blood without biopsy.

## Methods

### MiRNA microarray data

Serum miRNA data related to prostate cancer were acquired from GSE112264 expression profile data in GEO https://www.ncbi.nlm.nih.gov/geo/query/acc.cgi?acc=GSE112264), and tissue data were downloaded from TCGA (https://portal.gdc.cancer.gov/). The dataset GSE112264 was generated using the GPL21263 platform, comprising 809 prostate cancer samples for the tumor group and 241 negative prostate cancer and 41 non-cancer samples for the control group (Urabe et al., 2019). Then, we obtained tissue miRNA data from TCGA database containing 497 prostate cancer and 52 normal samples.

### Identification of PCa-related miRNAs

We preprocessed serum miRNA data from PCa patients and the control group in the GSE112264 dataset using the online tool GEO2R (http://www.ncbi.nlm.nih.gov/geo/geo2r/) (Barrett et al., 2013). EdgeR was used to analyze DE-miRNAs associated with PCa in the TCGA database (Robinson et al., 2010). We set adjusted the *p*-value < 0.05 and |fold change (FC)| ≥1 as screening thresholds. The common DE-miRNAs from GSE112264 and TCGA are illustrated in Venn diagrams (http://bioinfogp.cnb.csic.es/tools/venny/index.html) (Jia et al., 2021).

### Prediction of potential target genes of DE-miRNAs

The web tool miRTarBase (http://mirtarbase.mbc.nctu.edu.tw/php/index.php), a specialized collection of experimental evidence supporting the miRNA–mRNA network, was introduced to predict the target genes of the common DE-miRNAs from GSE112264 and TCGA (Hua et al., 2020).

### Functional and pathway enrichment analyses

GO and KEGG pathway enrichment analyses were processed for these filtered DEGs. GO was extensive in annotating genes, gene products, and sequences. Similarly, KEGG is an interactive dataset for biological explanation and functional analysis of genome sequences, conducted using the clusterProfiler package (Kanehisa et al., 2017). DAVID (http://david-d.ncifcrf.gov/) offers the functional annotation and pathway enrichment analysis on significant target genes (Dennis et al., 2003). A *p*-value < 0.05 was considered statistically significant.

### Construction of the protein–protein interaction network and identification of hub genes

The PPI network was constructed to illustrate the association among the screened genes using the Search Tool for the Retrieval of Interacting Genes/Proteins (STRING) (http://string-db.org). The PPI node pairs with a combined score ≥0.4 were considered significant and introduced into subsequent analysis. The hub genes were selected and illustrated according to degree using the CytoHubba plugin of Cytoscape software (version 3.6.3) (Szklarczyk et al., 2023).

### Target gene expression analysis based on the UALCAN database

The UALCAN database (http://ualcan.path.uab.edu/analysis.html) is a portal for evaluating protein-coding transcriptome data and survival analysis with data obtained from TCGA (Chandrashekar et al., 2022). In this study, we compared the expression of the top 10 genes associated with miR-146a-3p and miR-136-3p, respectively, between normal and primary tumor samples.

### Patients’ sample collection before blood and tissue sampling

We procured serum and tissue specimens from individuals diagnosed with prostate cancer (PCa) (n = 22) and benign prostatic hyperplasia (BPH) (n = 19) at the First Affiliated Hospital of University of South China. Initially, the blood samples were subjected to centrifugation at 3000 *g* for 10 min at 4 °C to isolate the serum. The supernatant was decanted, and the residual cellular debris was further eliminated through centrifugation at 3000 *g* for 10 min at 4 °C. Eventually, the serum samples were partitioned and preserved at −80 °C for subsequent processing (Wang et al., 2015).

### RNA isolation and qRT-PCR for clinical samples

The method for extracting total RNA from clinical samples and conducting qRT-PCR strictly followed the manufacturer’s guidelines (TaKaRa, Kusatsu, Japan). All procedures were conducted in triplicate. In accordance with the manufacturer’s recommendations, miRNA levels were normalized to the internal control (5S rRNA). Real-time quantitative PCR was performed using an ABI 7500 Detection System (Applied Biosystems, CA, United States). The 2^−ΔΔCt^ method was used to determine the relative expression of target genes. GAPDH or U6 served as the internal reference control. All primers were listed as follows: MiR-136-3p: forward, 5′-CAU CAU CGU CUC AAA U-3′ and reverse, 5′-GTG CAG GGT CCG AGG T-3′; U6: forward, 5′-TGC GGG TGC TCG CTT CGG CAG C-3′ and reverse, 5′-GTG CAG GGT CCG AGG T-3′.

### Statistical analysis

Statistical analysis was performed using the two-tailed Student’s t-test to assess statistical significance between the two experimental groups for clinical sample validation using SPSS v20.0. The correlation of the miRNA expression levels in serum and tissue was analyzed using Pearson correlation in GraphPad Prism 8.3.0. The area under the curve (AUC) and 95% confidence intervals (CIs) were calculated using ROC analysis with the pROC R package to evaluate the discriminatory power of the miRNAs in distinguishing the PCa group from the control group. Sensitivity was plotted against 1-specificity for the binary classifier. A *p*-value <0.05 was considered statistically significant.

## Results

### Identification of DE-miRNAs and target genes

**FIGURE 1:**
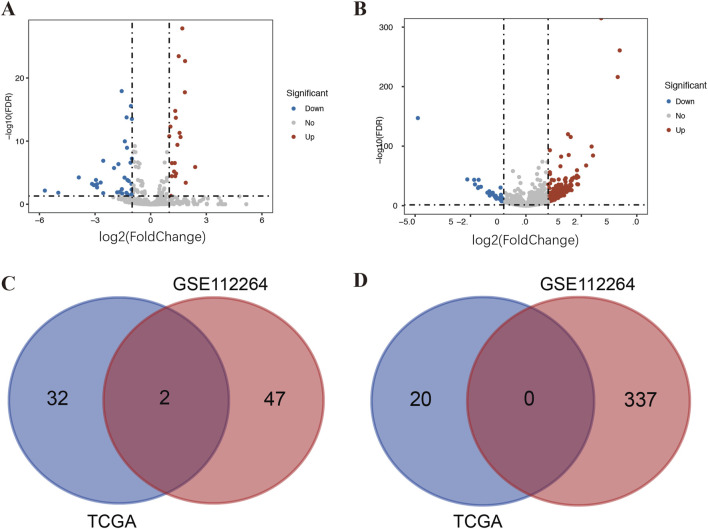
Identification of DE-miRNAs in serum and tissue samples of PCa patients. **(A)** DE-miRNAs between 497 prostate cancer and 52 normal tissue samples from TCGA; **(B)** DE-miRNAs between 809 prostate cancer and 282 control serum samples (including 241 negative prostate cancer and 41 non-cancer controls) from GSE112264; **(C)** Venn diagram of PCa-related downregulated DE-miRNAs in TCGA and GSE112264; and **(D)** Venn diagram of PCa-related upregulated DE-miRNAs in TCGA and GSE112264.

A total of 386 DE-miRNAs were screened out from the GSE112264 dataset, including 337 upregulated miRNAs and 49 downregulated miRNAs. A total of 54 DE-miRNAs, comprising 20 upregulated miRNAs and 34 downregulated miRNAs, were extracted from TCGA. For better visualization, the volcano plot and the Venn plot are presented in Figure 1. According to the adjusted *p*-value and logFC, miR-146a-3p and miR-136-3p (Table 1) were found to be the common downregulated miRNAs after the overlap of GSE112264 and TCGA. A total of 948 potential target genes were predicted for the two downregulated miRNAs through miRTarBase.

**TABLE 1:** PCa-related miRNAs overlapped in GSE112264 and TCGA.

miRNA ID	GSE112264		TCGA	
	log_2_FC	adj. P-value	log_2_FC	adj. P-value
has-miR-136-3p	-1.01	2.09E-09	-1.56	4.02E-03
has-miR-146a-3p	-1.02	2.33E-10	-1.67	1.14E-02

### Functional enrichment analysis

**FIGURE 2:**
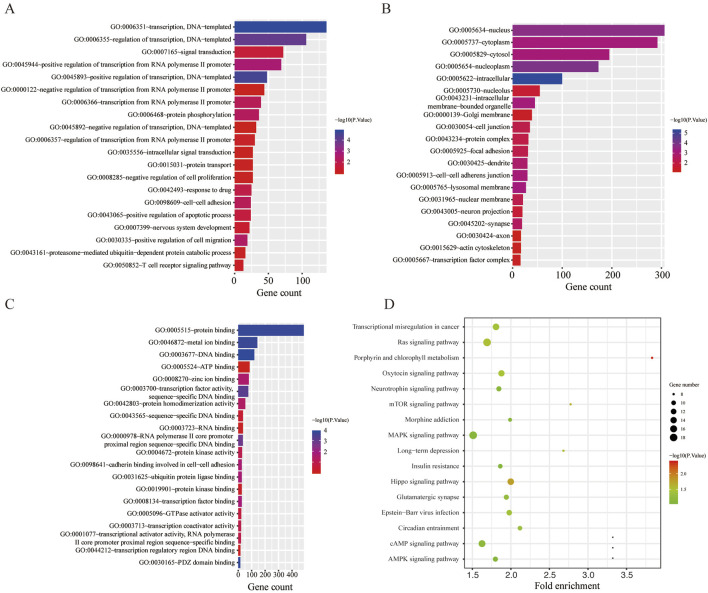
Functional enrichment analysis for the target genes of miR-136-3p and miR-146a-3p. **(A)** Enriched biological process (BP) of the downregulated miRNAs; **(B)** enriched cellular component (CC) of the downregulated miRNAs; **(C)** enriched molecular function (MF) of the downregulated miRNAs; and **(D)** KEGG pathway enrichment analysis of the downregulated miRNAs.

GO and KEGG functional annotation analyses were performed on these target genes mentioned above. The top 20 enriched GO items are listed in Figures 2A–C. Two GO terms from the category of biological process (BP) were enriched, including transcription and regulation of transcription. In terms of cellular components (CCs), downregulated DE-miRNAs were mainly enriched in the nucleus and cytoplasm. In the molecular function (MF) ontology, the most significant GO terms were protein binding. Additionally, three KEGG pathways were enriched for the downregulated genes, namely, porphyrin and chlorophyll metabolisms, the mammalian target of rapamycin (mTOR) signaling pathway, and long-term depression. The detailed results are presented in Figure 2D.

### Construction of the protein–protein interaction network and identification of hub genes

**FIGURE 3:**
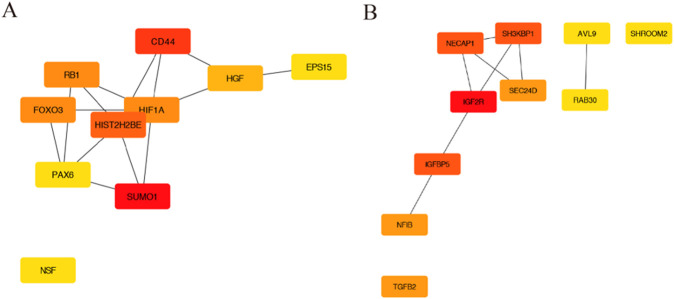
The PPI network construction and hub genes. **(A)** PPI network for miR-146a-3p; **(B)** PPI network for miR-136-3p.

The PPI network was constructed using STRING, and then, a total of 853 nodes and 3,370 edges were mapped in the PPI network of miR-146a-3p and miR-136-3p. The combined scores higher than 0.4 in PPIs were used for constructing the PPI networks. The CytoHubba plugin was used to analyze and visualize the top 10 genes, as shown in Figure 3.

### Hub gene expression in PCa using the UALCAN database

**FIGURE 4:**
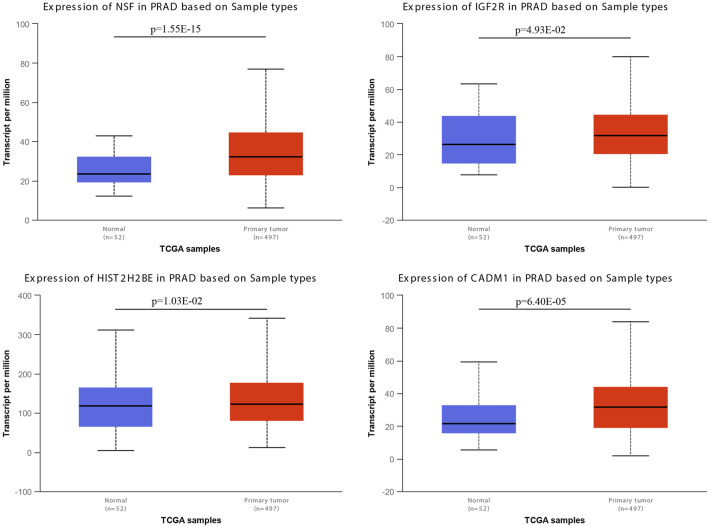
mRNA expressions of NSF, HIST2H2BE, IGF2R, and CADM1 from the UALCAN database.

We examined the expression levels of miR-146a-3p and miR-136-3p in PCa using the UALCAN database, and the results are shown in Figure 4 and Table 2. For miR-146a-3p, NSF and HIST2H2BE in PCa tissues were significantly increased compared with normal tissues, while CD44, H1F1A, PAX6, and RB1 showed the reverse tendency. For miR-136-3p, IGF2R and CADM1 were significantly elevated in PCa tissues, while NF1B, TGFB2, and SNTB2 were significantly downregulated. It is well known that miRNAs negatively regulate target genes at the transcriptional level. Therefore, the significantly upregulated genes (NSF, HIST2H2BE, IGF2R, and CADM1) could be potentially modulated through miR-146a-3p and miR-136-3p.

**TABLE 2:** *p*-value of the top 10 hub genes for miR-146a-3p and miR-136-3p from the UALCAN database.

miR-146a-3p			miR-136-3p		
Gene symbol	Degree	*p*-value	Gene symbol	Degree	*p*-value
SUMOI	33	7.94E-01	IGF2R	4	4.93E-02
CD44	32	1.44E-03	NECAPI	3	9.27E-02
HIST2H2BE	31	1.03E-02	SH3KBPl	3	1.50E-01
HIFIA	29	6.82E-03	IGFBP5	3	1.48E-02
FOX03	29	2.10E-01	NFIB	2	5.22E-03
RBI	29	2.43E-06	SEC24D	2	7.54E-01
HGF	28	2.99E-01	TGFB2	2	9.62E-04
PAX6	27	4.47E-05	CADMI	1	6.40E-05
NSF	27	1.55E-15	SNTB2	1	4.44E-04
EPS15	27	6.14E-01	SHROOM2	1	1.61E-01

### Validation of miR-146a-3p and miR-136-3p expressions in GEO, TCGA, and clinical samples

**FIGURE 5:**
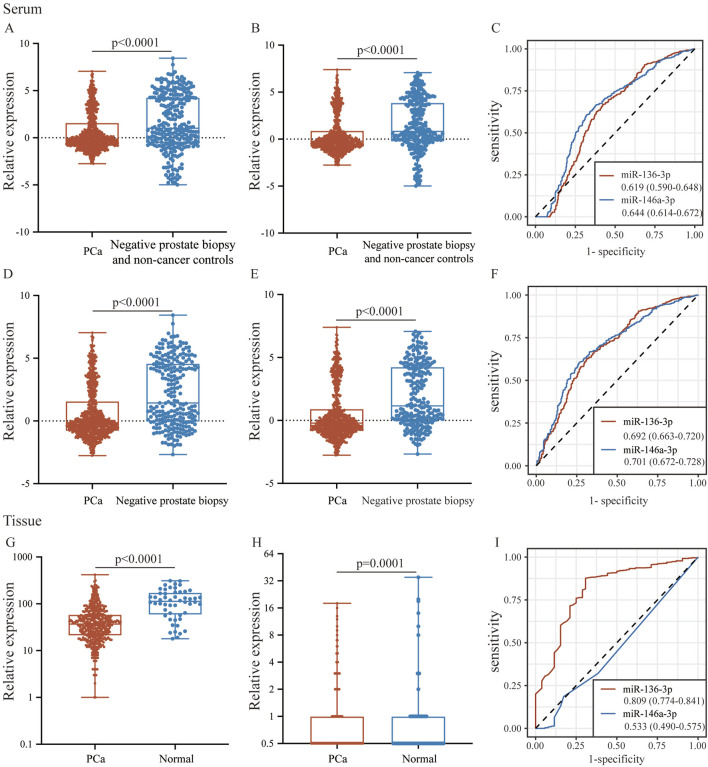
The expression and potential identification of miR-136-3p and miR-146a-3p in serum and tissue samples from the public database. **(A,B)** The expressions of miR-136-3p and miR-146a-3p in serum between the PCa group and the negative prostate biopsy and non-cancer group; **(C)** the potential of miR-136-3p and miR-146a-3p in serum for the identification of PCa from the GSE112264 dataset; **(D,E)** the expressions of miR-136-3p and miR-146a-3p in serum between the PCa group and the negative prostate biopsy group; **(F)** the potential of miR-136-3p and miR-146a-3p in serum for the identification of PCa excluding non-cancer patients; **(G,H)** the expressions of miR-136-3p and miR-146a-3p in tissue between the PCa group and the normal group; and **(I)** the potential of miR-136-3p and miR-146a-3p in tissue for the identification of PCa from TCGA.

**FIGURE 6:**
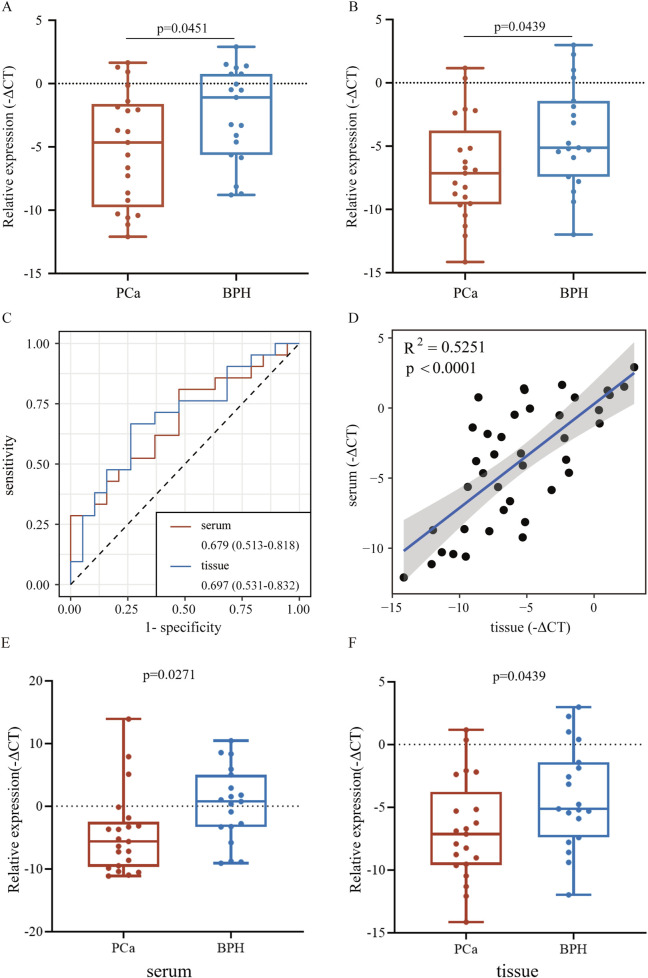
Relative expression of miR-136-3p in serum and tissue from clinical samples using qRT-PCR. **(A,B)** Expression of miR-136-3p in serum and tissue samples from PCa and BPH patients measured using qRT-PCR; **(C)** the potential of miR-136-3p for the identification of PCa from clinical serum and tissue samples; **(D)** the correlation of expression of miR-136-3p in serum and tissue samples; and **(E,F)** the expression of miR-146a-3p in serum and tissue samples from PCa and BPH patients measured using qRT-PCR.

To validate the consistency of expression levels in serum and tissue samples, we compared the expression of the downregulated miRNAs (miR-146a-3p and miR-136-3p) using a public database. As shown in Figure 5, miR-146a-3p and miR-136-3p were significantly downregulated in the PCa serum sample compared with the negative prostate biopsy and non-cancer patients. Similarly, the same tendency was found after excluding the non-cancer sample from the control group. In the TCGA database, miR-136-3p was confirmed to be markedly downregulated in PCa tissue samples, but a similar tendency for miR-146a-3p was not observed, as indicated by its poor AUC. The diagnostic potential of miR-146a-3p and miR-136-3p in PCa was assessed by plotting ROC curves with 95% CI. In serum samples, the AUC values of miR-146a-3p and miR-136-3p for distinguishing the PCa group from the control group were 0.644 (95% CI: 0.614–0.672) and 0.619 (95% CI: 0.590–0.648), respectively. Furthermore, when only negative prostate biopsy samples were chosen as the control group, the AUC values of miR-146a-3p and miR-136-3p were 0.701 (95% CI: 0.672–0.728) and 0.692 (95% CI: 0.663–0.720), respectively. For tissue samples, the AUC of miR-136-3p still showed a high level of 0.809 (95% CI: 0.774–0.841), while that of miR-146a-3p decreased to 0.533 (95% CI: 0.490–0.575). Detailed data are shown in Table 3. The different results of miR-146a-3p in serum and tissue samples suggested that miR-146a-3p might not be a reliable biomarker. We further examined miR-136-3p expression in clinical PCa and BPH samples using qRT-PCR, and the baseline characteristic is listed in Table 4. As shown in Figure 6, miR-136-3p is significantly downregulated both in PCa serum and tissue samples. The Spearman correlation test also confirmed the positive correlation between the expression in serum and tissue. We also examined the expression levels of miR-146a-3p in serum and tissue; both serum and tissue samples showed significant differences between PCa and BPH patients, while correlation analysis revealed no significant association between serum and tissue (R^2^<0.001; *p* = 0.9116). Overall, these results suggested that miR-136-3p could serve as a clinical diagnostic biomarker for PCa using only a blood sample.

**TABLE 3:** Biomarker indices of miR-136-3p and miR-146a-3p from serum and tissue databases using the ROC curve.

Group	miRNA	AUC (95% CI)	Sensitivity (95% CI)	Specificity (95% CI)	Youden index	Best cut-off	*p*-value
PCa vs negative prostate biopsy and non-cancer group	miR-136-3p	0.619 (0.590-0.648)	66.87 (63.5-70.1)	57.8 (51.8-63.6)	0.2467	0.557	<0.0001
	miR-146a-3p	0.644 (0.614-0.672)	66.63 (63.3-69.9)	62.06 (56.1-67.7)	0.2868	0.302	<0.0001
PCa vs negative prostate biopsy and non-cancer group	miR-136-3p	0.692 (0.663-0.720)	62.67 (59.2-66.0)	68.88 (62.6-74.7)	0.3115	0.302	<0.0001
	miR-146a-3p	0.701 (0.672-0.728)	60.37 (57.1-64.0)	73.44 (67.4-78.9)	0.3401	0.072	<0.0001
TCGA	miR-136-3p	0.809 (0.774-0.841)	87.68 (84.5-90.4)	69.23 (54.9-81.3)	0.5691	92	<0.0001
	miR-146a-3p	0.533 (0.490-0.575)	98.59 (97.1-99.4)	11.54 (4.4-23.4)	0.1012	7	0.3801

**TABLE 4:** Baseline characteristics of patients; N represents the number.

Characteristic	Number (%)
All patients, N	21
Age, years, n (%)	
<60	9 (42.9)
≥60	12 (57.1)
PSA, ng/mL, n (%)	
<4	5 (23.8)
≥4	16 (76.2)
Gleason score, n (%)	
≤7	13 (61.9)
>7	8 (38.1)
Pathologic stage, n (%)	
T2	11 (52.4)
T3	10 (47.6)

## Discussion

PCa claims thousands of lives every year, mainly due to its drug resistance and invasiveness, despite multiple new drug approvals in recent years. The early diagnoses of PCa have become a very important issue. Liquid biopsy is a scientific source of biomarkers and is currently a major focus of clinical research. This approach can provide direct assistance for disease diagnosis using blood, urine, and other body fluids and allows for sustainable monitoring of the disease’s response to treatment (Raza et al., 2022; Detassis et al., 2024; Sequeira et al., 2023; Mao et al., 2023; Vaidyanathan et al., 2016). However, the heterogeneity of biomarkers in serum and tissue remains to be considered. MiRNAs play a critical role in the regulation of cancer progression and serve as biomarkers at various stages of PCa. Scientists have reported that inhibiting miR-4719 and miR-6756-5p to upregulate IL-24 may represent a therapeutic strategy for aggressive PCa (Das et al., 2019).

In this study, we aimed to identify potential biomarkers for PCa by detecting miRNAs from serum and tissue datasets. The differential expression analysis was performed using a public database. Two miRNAs, miR-146a-3p and miR-136-3p, both downregulated in serum and tissue, were identified. Recent research suggested that miR-146a-3p is related to the occurrence and progression of diseases, including asthma, allergic rhinitis, and Paget’s disease (Duan et al., 2023; Xia et al., 2023; Stephens et al., 2020). Similarly, miR-136-3p is also reported to inhibit tumorigenesis (Xu, 2020). However, it has not been reported that miR-146a-3p and miR-136-3p may participate in the progression of PCa. It is significant to explore the functions of miR-146a-3p and miR-136-3p in PCa and elucidate their mechanisms. Next, the researchers predicted 948 target genes that might be regulated using the two common downregulated miRNAs and performed functional enrichment analysis. The results demonstrated that these target genes were enriched in protein binding, porphyrin and chlorophyll metabolisms, and the mTOR signaling pathway.

To our knowledge, numerous RNA-binding proteins are involved in regulating the post-transcriptional processes and have a profound impact on RNA metabolism (Corley et al., 2020). It has been well documented that protein binding is closely associated with tumor migration and invasion (Wang et al., 2022). The activation of the mTOR pathway is the major promoter of various cellular activities, including protein synthesis, tumor proliferation and invasion, autophagy, and cellular metabolism (Hua et al., 2019; Mossmann et al., 2018). Recent studies have indicated that the complex interactions within the PI3K–AKT–mTOR pathway may promote PCa progression and influence the resistance of tumor cells to mTOR-targeted therapy (Dai et al., 2021).

According to the PPI network, the top 10 hub genes of two downregulated miRNAs were screened out. With further evaluation using the UALCAN database, we found that NSF, HIST2H2BE, IGF2R, and CADM1 were identified as hub genes with higher degrees. A few studies have reported these genes in other diseases and cancers. N-ethylmaleimide-sensitive factor (NSF) is an ATPase involved in intracellular vesicle trafficking, mostly found in eukaryotic cells, and is, therefore, considered a potential therapeutic target (Calvert et al., 2007). HIST2H2BE was demonstrated to regulate cancer progression and development. The upregulation of HIST2H2BE has been found in gastric cancer and invasive ductal carcinoma (Guo et al., 2010; He et al., 2021). However, the association with PCa still needs to be investigated. IGF2R, the hub gene predicted in this study, functions as a receptor for insulin-like growth factor 2. Apart from the intracellular trafficking of lysosomal enzymes, mutation or loss of this gene has been confirmed to be associated with various cancers, including gastrointestinal cancer, renal tumors, and osteosarcoma (Oates et al., 1998; Xu et al., 1997; Broqueza et al., 2021). However, the underlying mechanism by which IGF2R promotes the progression of PCa, especially its crosstalk with microRNA, remains unsolved. The final predicted gene, CADM1, is also involved in several processes, including cell recognition, positive regulation of cytokine secretion, and natural killer cell-mediated susceptibility to cytotoxicity (Li et al., 2021). The functions of these hub genes encompass intracellular transport (NSF), epigenetic regulation (HIST2H2BE), growth factor signaling regulation (IGF2R), and cell adhesion (CADM1), all of which are core biological processes closely associated with cancer development and progression, including tumor cell proliferation, survival, invasion, and metastasis. It is generally assumed that miRNAs negatively regulate their target genes, but the specific regulatory pathway remains to be investigated.

Then, we validated the expression and prognostic roles of miR-146a-3p and miR-136-3p in the public database and clinical PCa patients. Only miR-136-3p was downregulated both in serum and tissue samples according to GEO and TCGA. A consistent trend was confirmed through qRT-PCR analysis of clinical samples. The observed consistency raises the question of whether blood-based assessment of miR-136-3p could replace biopsies for early diagnosis. Compared to the traditional biomarker such as PSA, the clinical applicability of miR-136-3p needs further rigorous experiments and clinical trials. However, miR-146a-3p heterogeneity in serum versus tissue compels us to reflect on the underlying reasons. Previous studies have also found inconsistency; Skog et al. (2008) detected tumor-specific miRNAs in the serum of patients with glioblastoma, but tissue expression levels were not fully correlated with those in serum. The miRNAs in the multi-source serum of circulating miRNAs may originate from organs other than tumor tissue (such as liver and immune cells) or extracellular vesicles (exosomes and microparticles), and miRNAs in tissue samples more directly reflect the local microenvironment (Turchinovich et al., 2011). Valadi et al. (2007) confirmed that extracellular vesicles can transfer miRNAs from donor cells to recipient cells, resulting in incomplete consistency between circulating miRNAs and tissue sources. Non-tumor cells, such as tumor-associated fibroblasts (CAFs) and immune cells, may secrete specific miRNAs into the bloodstream, while miRNAs in tissue samples mainly come from tumor cells themselves. Researchers found that breast cancer cells secrete miRNA through exosomes, but CAFs also contribute to the circulating miRNA (Melo et al., 2014). Disease stages and dynamic changes may also contribute to the secretion mode of miRNA. Early-stage tumors may preferentially secrete specific miRNAs into the bloodstream (such as miR-21 as an early diagnostic marker), while late-stage tumor tissues may experience changes in miRNA release patterns due to necrosis (Chen et al., 2008).

In conclusion, we confirmed that miR-136-3p is poorly expressed in PCa serum and tissue samples and might serve as a biomarker in PCa. However, our study has some limitations: (1) only two target genes with overlapping downregulated miRNAs were identified for further enrichment analysis; (2) the hub genes of miR-136-3p showed low degree, and detailed molecular mechanisms of miR-136-3p downregulation in PCa patients are lacking; (3) more clinical survival data need to be included for detecting prognosis efficiency; and (4) why miR-146a-3p shows a different tendency between serum and tissue remains to be investigated.

## Conclusion

In our study, we confirmed that miR-136-3p plays an important role in the progression of PCa through bioinformatics analysis and qRT-PCR validation. These findings provide new approaches for targeting miR-136-3p as a biomarker of PCa.

## Data Availability

The datasets presented in this study can be found in online repositories. The names of the repository/repositories and accession number(s) can be found in the article/supplementary material.
