# Supplementary figures and images for: Systematic characterization of cross-source miRNA biomarkers in prostate cancer with computational-experimental integrated analysis
Source: Front Cell Dev Biol. 2025 Sep 25;13:1605297. doi: 10.3389/fcell.2025.1605297 (PMC12507830; doi:10.3389/fcell.2025.1605297)

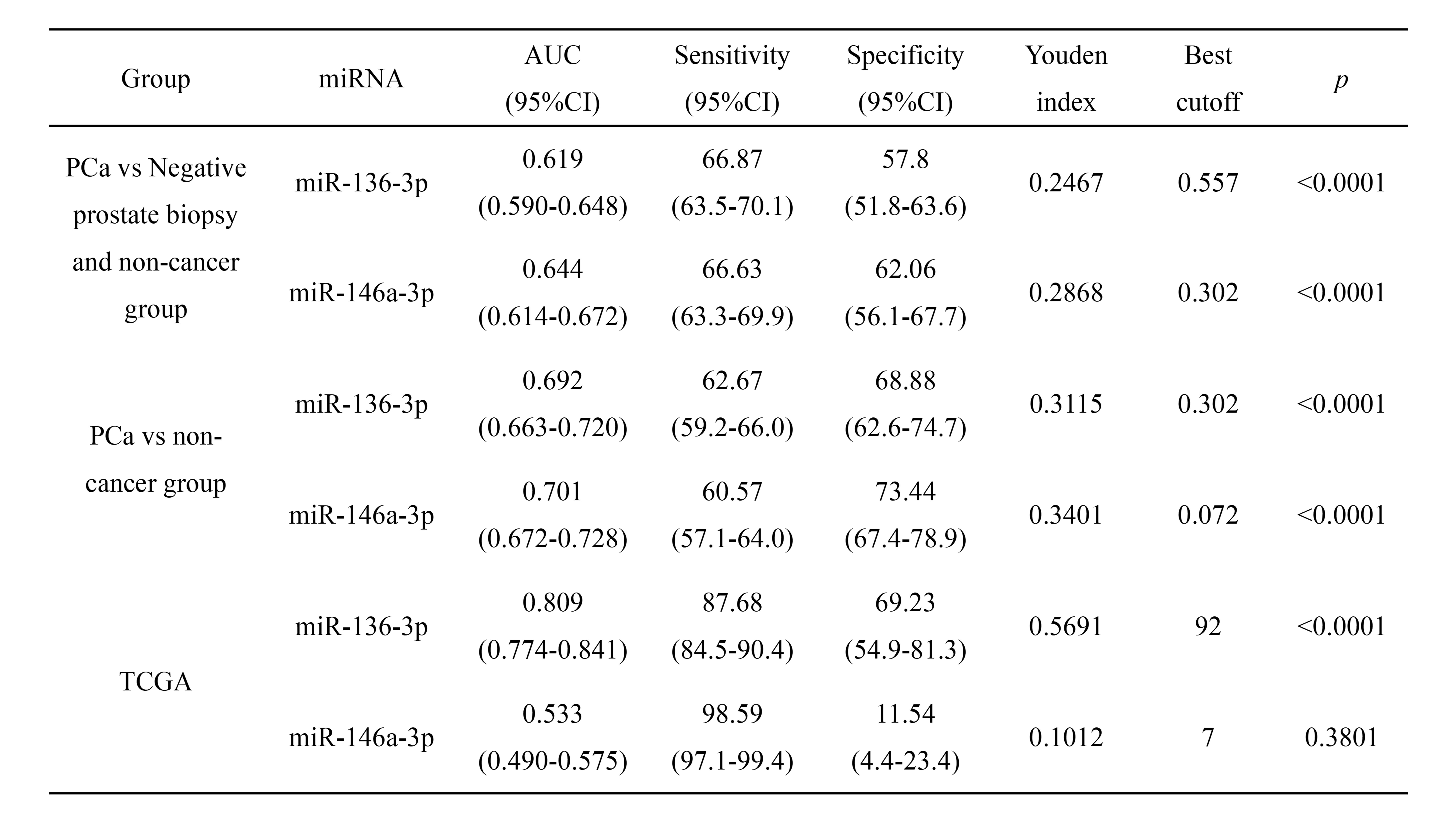

Supplement: Supplementary file 1 [file Image3.tif]

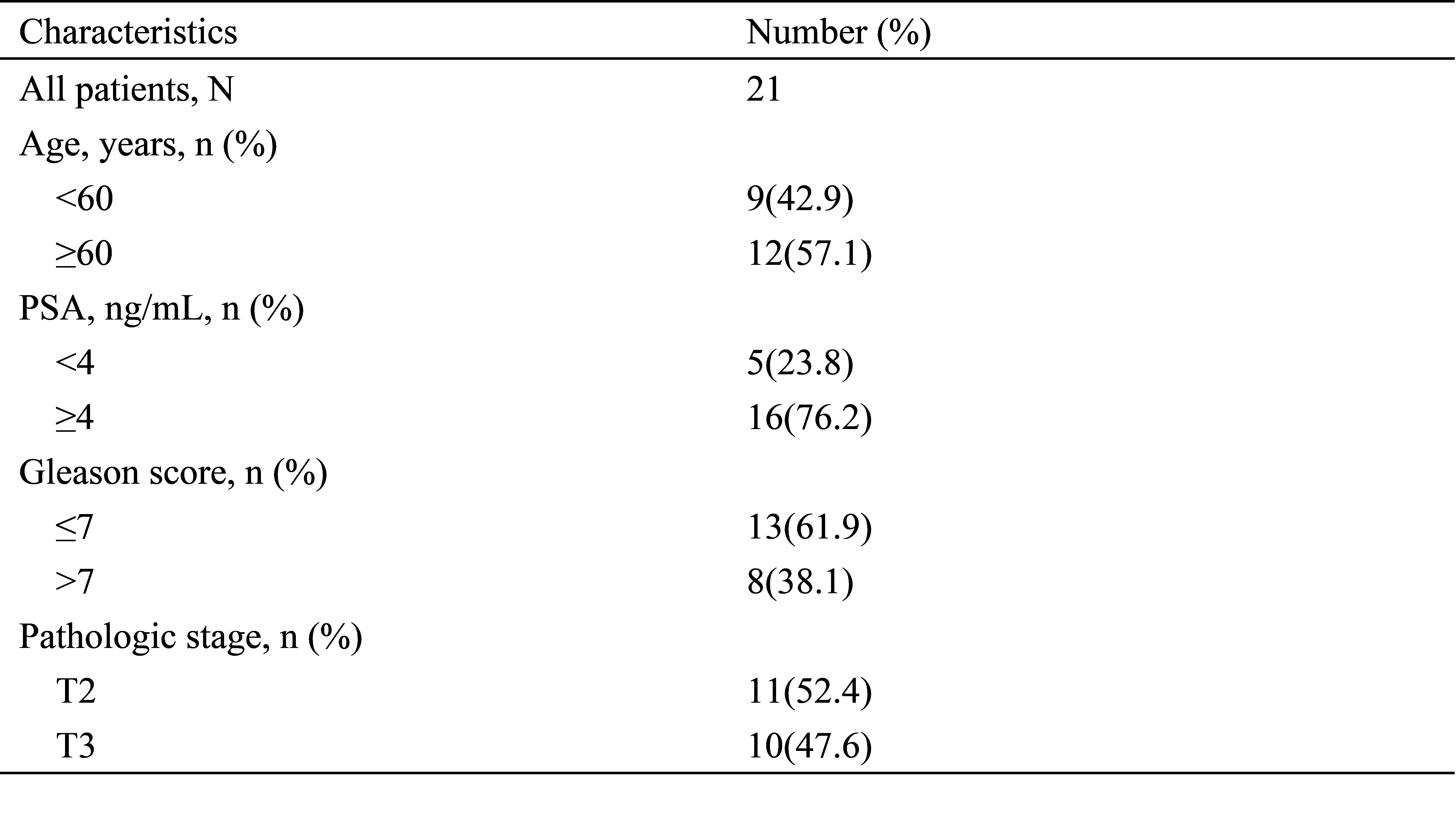

Supplement: Supplementary file 2 [file Image4.tif]

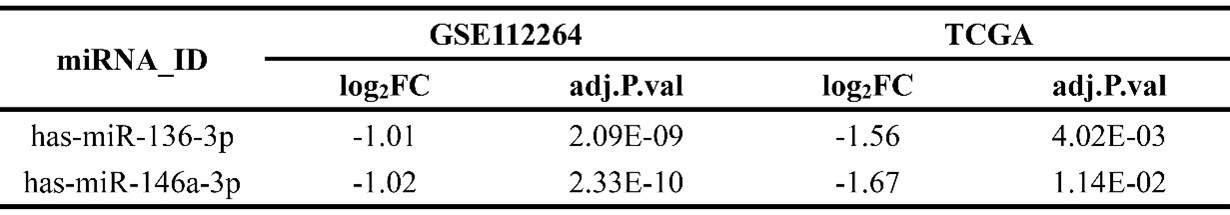

Supplement: Supplementary file 3 [file Image1.jpeg]

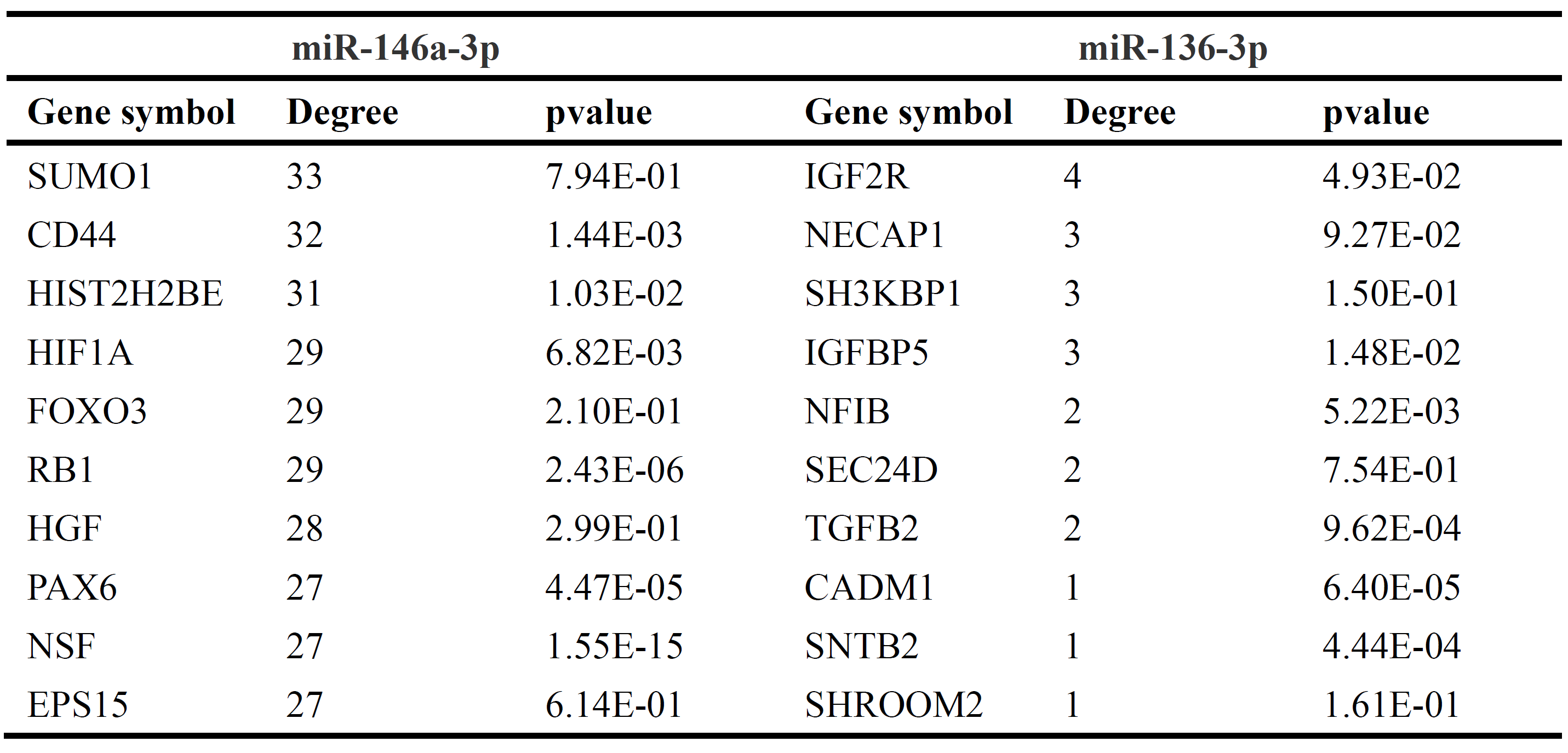

Supplement: Supplementary file 4 [file Image2.jpeg]
